# A GIS-based AHP approach integrating geospatial and magnetic data for groundwater potential mapping in a structurally complex arid region, Egypt

**DOI:** 10.1038/s41598-026-52393-y

**Published:** 2026-05-18

**Authors:** Sara Zamzam, Ethaar Gadallah, Ahmed Henaish, Ahmed M. Nosair

**Affiliations:** 1 Geology Department, Faculty of Science, Zagazig University, P.O. Box: 44519, Zagazig, Egypt; 2 Environmental Geophysics Lab (ZEGL), Geology Department, Faculty of Science, Zagazig University, P.O. Box: 44519, Zagazig, Egypt

**Keywords:** Arid coastal regions, Groundwater potentiality, Analytical hierarchy process, Geospatial data analysis, Structural controls, Sustainable development, Environmental sciences, Hydrology, Solid Earth sciences

## Abstract

Groundwater exploration in arid regions of the Eastern Desert of Egypt requires an integrated understanding of the structural, geomorphic, and subsurface controls governing recharge and storage. This study presents a GIS-based groundwater potentiality model for a structurally complex rift-related zone along the southern Esh El Mellaha Block, between the Gulf of Suez and northern Red Sea. Sixteen topographical, meteorological, hydrological, and surface geological factors were systematically integrated with particular magnetic basement-depth modeling and subsurface fault architecture, which were weighted using the Analytical Hierarchy Process (AHP). Results demonstrate that regional tectonic configuration and structural geometry, rather than surface geomorphic factors alone, exert the primary control on groundwater distribution. High-potential zones are concentrated within major structural lows, including the Tarboul syncline, West Hurghada trough, and El Gouna fan system, where thick Quaternary deposits and enhanced infiltration prevail. The ENE-trending Bali Shear Zone acts as a key conduit for focused recharge by enhancing fracture permeability and linking the Gulf of Suez and Red Sea structural domains. Model validation yielded an AUC of 0.80, with balanced sensitivity and specificity (0.74), indicating reliable predictive performance. Single parameter sensitivity analysis confirms the robustness of the model and indicates that structural and geological factors exert the strongest control on groundwater potential distribution. The study emphasizes the role of structural architecture in groundwater assessment and supports future sustainable water-resource development in arid extensional tectonic settings.

**Supplementary Information:**

The online version contains supplementary material available at 10.1038/s41598-026-52393-y.

## Introduction

**Fig. 1:**
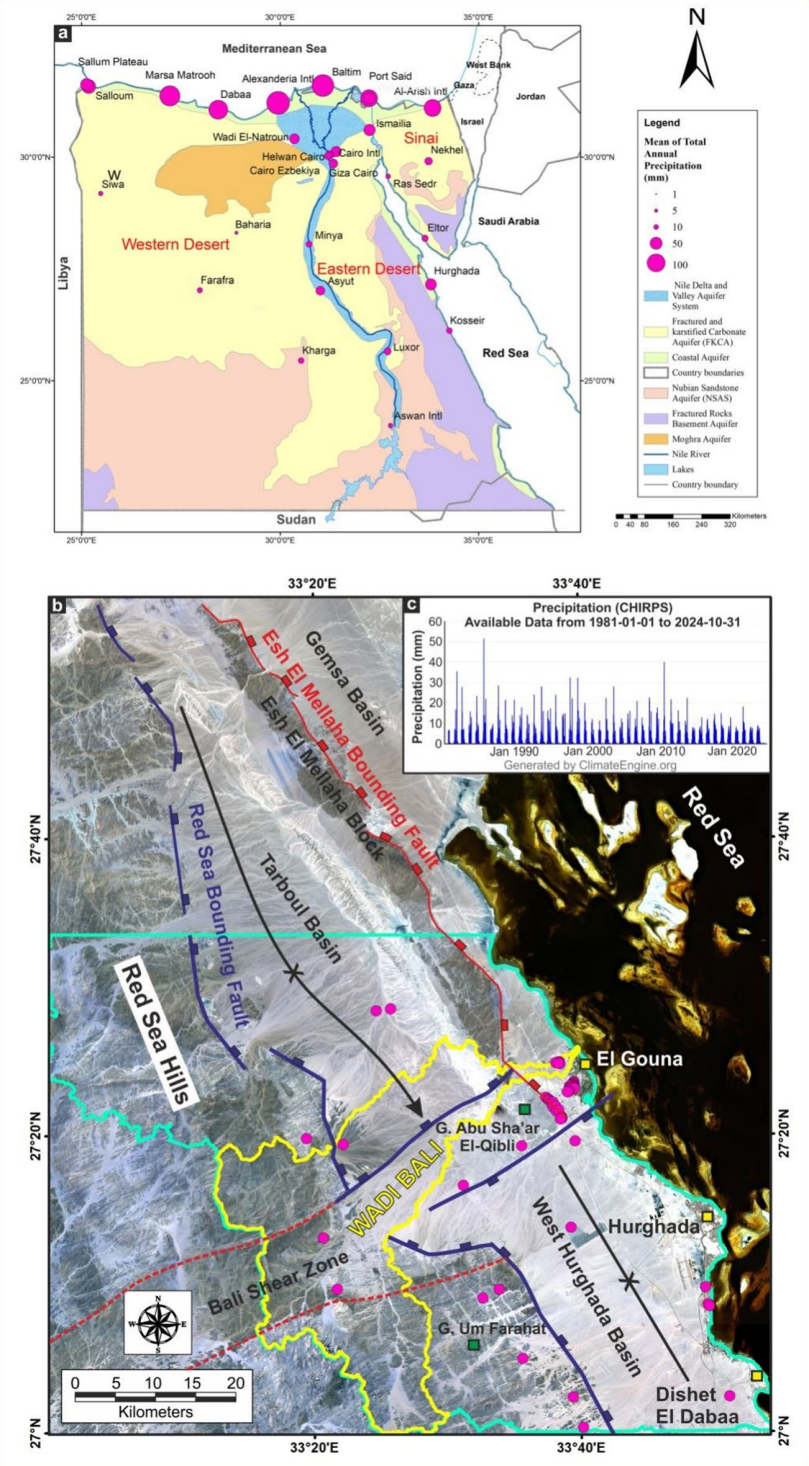
(**a**) The distribution of aquifer systems and mean annual precipitation in Egypt^15–17^, highlighting regional water availability contrasts; (**b**) Landsat image shows the main structural elements at the northwestern Red Sea and Esh el Mellaha block. Magenta circles represent well locations. Green polygon represents the study area; (**c**) The available daily precipitation data of the study area over 43 years shows the fluctuation in rainfall rates. (Generated by ArcGIS v.10.8. https://www.esri.com/ en- us/arcgis/, and CorelDraw X5, https://www.coreldraw.com).

Water scarcity represents one of the most critical environmental challenges in arid and semi-arid regions, where limited and irregular precipitation places increasing pressure on available water resources. Egypt is classified among countries experiencing high water stress, with freshwater withdrawals representing nearly 40% of its total renewable water supply^1^. This situation is largely driven by the country’s strong dependence on the Nile River as the primary surface-water source, while contributions from rainfall, flash floods, and groundwater remain limited and spatially uneven^2^. Figure 1a illustrates the distribution of Egypt’s major aquifer systems and highlights the strong contrast between water availability and regional precipitation patterns. Under ongoing climatic variability, changes in the frequency, intensity, and spatial distribution of rainfall further complicate sustainable water-resource planning^3^.

The Eastern Desert of Egypt exemplifies these challenges. Annual rainfall rarely exceeds 25 mm and is mainly confined to coastal and northern sectors^4^. Although rainfall events are infrequent, they are often torrential, generating flash floods that promote focused recharge along wadis, alluvial fans, fractured basement terrains, and structurally controlled basins. Previous studies estimate that approximately 21–31% of rainfall may infiltrate through such localized recharge zones^5^. These observations emphasize the importance of identifying structural pathways and geomorphic settings that enhance groundwater recharge in arid environments.

The present study focuses on a structurally complex area along the northwestern margin of the Red Sea covering approximately 3504.66 Km^2^ between latitudes 26°58’56”−27°33’38” N and longitudes 32°59’−33°54’39” E (Fig. 1b). The area is centered on Wadi Bali, an active drainage basin frequently affected by flash-flood events, indicating significant episodic recharge potential. It also includes the rapidly expanding coastal cities of Hurghada and El Gouna. El Gouna was designated by UNEP in 2014 as the first “Global Green Town” in Africa and the Middle East due to its sustainability initiatives. Rapid urban and touristic development, combined with limited water resources, has significantly increased local water demand. In El Gouna, for instance, approximately 945,000 m² of landscaped areas contribute to elevated water consumption^6^. Figure 1c highlights the strong temporal variability of rainfall in the study area over the past four decades, further underscoring the need for reliable local groundwater resources.

Recent studies focused on natural resource exploration, particularly groundwater and mineral deposits, increasingly depend on integrating diverse datasets and analytical techniques rather than relying solely on traditional approaches, as this integration enhances the reliability of interpretations and supports informed decision-making^7–10^. Techniques such as GIS-based modeling, remote sensing, and both knowledge-driven and data-driven approaches have been widely applied for groundwater exploration^11–14^. Knowledge-driven methods such as AHP rely on expert judgment, theoretical rules, and offer transparency, as well as being suitable for data-scarce regions. On the other hand, data-driven ML approaches such as RF and ANN learn relationships directly from empirical data. They often achieve higher predictive accuracy when sufficient data are available, but with reduced interpretability. Accordingly, alternative weighting schemes and modeling frameworks may yield different spatial outcomes in assessing groundwater potential.

In the Eastern Desert aquifer systems (Fig. 1a), faults, shear zones, fracture networks, and rift-related structures play a primary role in enhancing secondary porosity and permeability, thereby governing recharge pathways and groundwater storage. This control is particularly significant in extensional settings and rift-related structures, which remain underrepresented in groundwater modeling literature. Moreover, the limited integration of both surface-derived factors (e.g., drainage, slope, geomorphology, lithology, and lineaments) and subsurface geophysical constraints into groundwater potential assessments represents a key research gap.

In this context, the present study develops an integrated GIS-based AHP framework for groundwater potential assessment for the northwestern Red Sea margin of the Eastern Desert, Egypt. Unlike conventional groundwater potential studies that rely primarily on surface geological and geomorphological indicators, this research incorporates subsurface geophysical constraints, including magnetic basement-depth modeling and structurally interpreted fault systems, as key conditioning factors controlling groundwater occurrence. The approach evaluates the role of tectonic architecture in regulating groundwater recharge and storage within fractured basement aquifers associated with rift-related extensional structures. The resultant model is validated using well data and sensitively analyzed by the single parameter method to provide a transferable methodological framework for groundwater exploration and sustainable water-resource management in arid, structurally complex regions.

## Regional geology and structural setting

Understanding the regional geology and structural framework of the study area is essential for evaluating groundwater accumulation, particularly in terrains where tectonics exert a strong influence on surface and subsurface hydrology. The investigated region occupies the southern part of the Esh El Mellaha Block along the northwestern margin of the Red Sea and the southwestern margin of the Gulf of Suez rift system (Fig. 1b).

The structural development of the region is linked to the Oligocene-Miocene development of the Red Sea-Gulf of Suez rift system. Initial rifting during the Late Oligocene–Early Miocene produced large NE-dipping normal faults and half-graben basins (asymmetric extensional basins bounded by a major normal fault). Continued extension during the Middle Miocene resulted in block rotation, the formation of accommodation or transfer zones (structural corridors that transfer strain between fault segments), and localized deposition of carbonates and evaporites. Late-stage deformation involved the reactivation of older faults, strike-slip adjustments along NE-SW corridors, and uplift and erosion of rift shoulders. This multi-phase tectonic history generated a complex structural architecture that continues to control drainage organization, sediment accumulation, and hydrogeological compartmentalization.

The Esh El Mellaha Block stands as a prominent, rugged highland composed mainly of Precambrian crystalline basement rocks belonging to the northern Arabian-Nubian Shield^18^. These rocks include granitic and metavolcanic assemblages intruded by mafic and felsic dikes. Basement exposures are intensely jointed and fractured, reflecting a protracted deformation history and creating structural heterogeneities that significantly enhance secondary porosity and permeability and influence groundwater storage and movement.

**Fig. 2:**
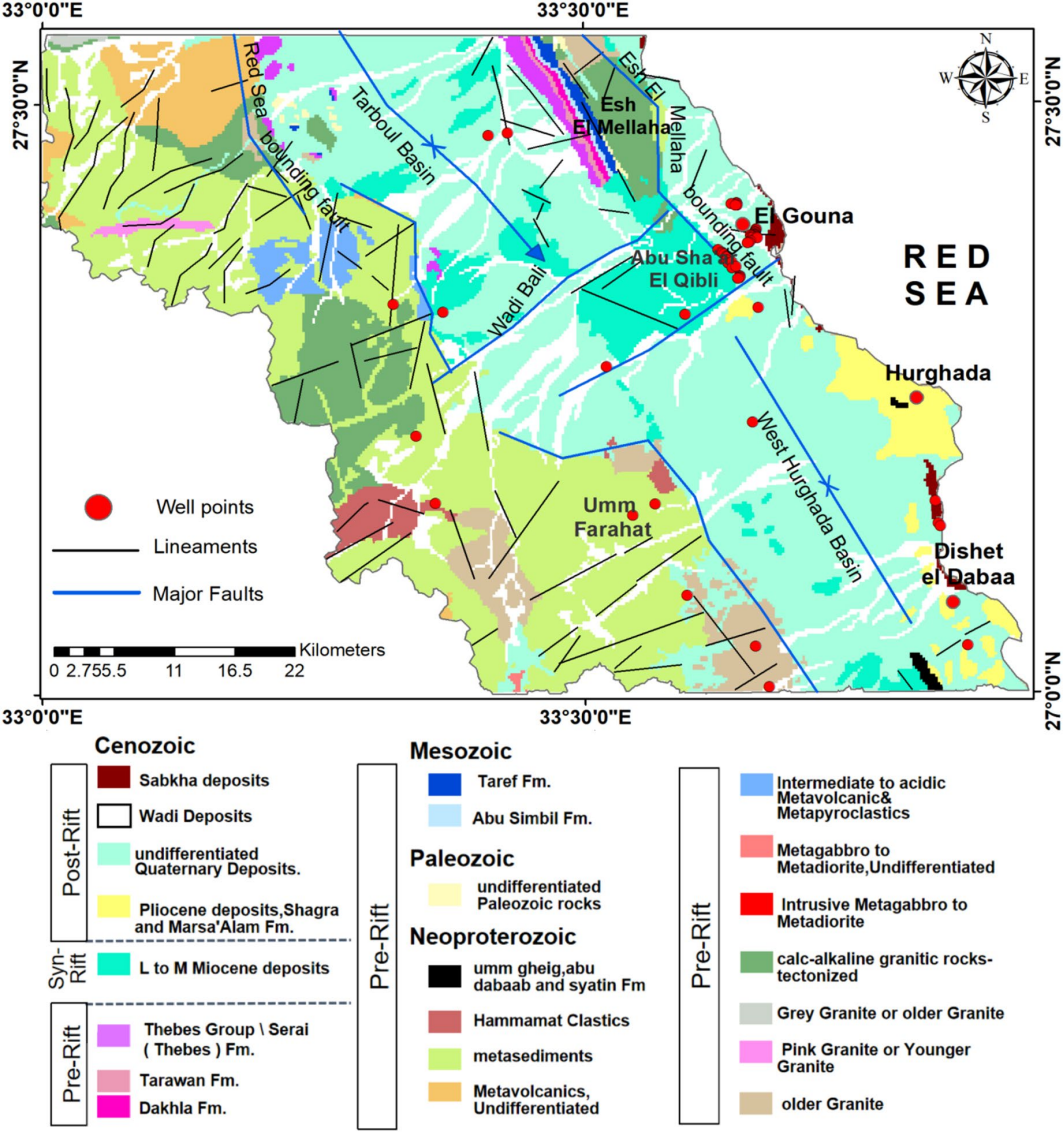
Geological map of the study area (modified after^23^. (Generated by ArcGIS v.10.8. https://www.esri.com/ en- us/arcgis/).

Miocene syn-rift sedimentary successions unconformably overlie the tilted basement blocks. Around Wadi Bali and its southern surroundings, the succession comprises reefal carbonates, clastic sandstones, and evaporitic units formed within extensional basins during active Gulf of Suez rifting (Fig. 2). Quaternary deposits form the youngest units and consist of alluvial fans, wadi-fill gravels, and coarse detrital sediments that host the shallow aquifers within the main drainage systems.

Structurally, the Esh El Mellaha Block forms the southwestern margin of the Gulf of Suez rift and is dissected by a network of rift-parallel NW-SE normal faults, together with NE-SW and N-S oriented lineaments that act as transfer zones between fault segments^19,20^. The principal Esh El Mellaha Boundary Fault is a steep, NE-dipping normal fault that accommodated significant displacement and controlled basement block tilting. These intersecting fault systems subdivide the region into smaller rotated panels, producing stepped topography and exerting strong control on drainage patterns, including the course of Wadi Bali.

Joint and fracture systems are particularly well developed at Gebel Abu Sha’ar El-Qibli and along the Esh El Mellaha ridge. Their geometry reflects multiple tectonic stress regimes and records the cumulative effects of Phanerozoic deformation^21^. These joints and fractures enhance secondary porosity within both basement and carbonate units, improving localized groundwater storage.

Two major regional fault systems further define the structural framework (Fig. 1b). The Esh El Mellaha Boundary Fault marks the eastern margin of the block and separates it from the Gemsa Basin, which is filled with syn- and post-rift Miocene sediments. The Red Sea Hills Bounding Fault, a major NW- to NNW-trending normal fault, defines the eastern edge of the Red Sea Hills, which consist entirely of Precambrian crystalline rocks dissected by ENE-trending wadis. Many of these wadis follow inherited structural fabrics, highlighting the role of pre-existing weaknesses in controlling surface runoff and groundwater recharge.

Between these two major faulted margins lies the Tarboul Basin (Fig. 1b), a SE-plunging synclinal structure that exposes a mixture of pre-rift and syn-rift units. This basin represents a major structural low that promotes sediment accumulation and provides favorable conditions for groundwater storage. To the south, the West Hurghada Basin forms a structurally controlled trough bounded by NW-SE and NNW-SSE normal faults. It represents an actively subsiding compartment that receives sediments from adjacent highlands and contributes to the regional structural asymmetry between the Gulf of Suez and the northern Red Sea.

A key structural element within the study area is the Bali Shear Zone, an ENE-trending structural corridor that aligns with the main course of Wadi Bali. Located between the Tarboul Basin to the northwest and the West Hurghada Basin to the southeast, this zone likely functioned as a rift-transfer zone during Gulf of Suez rifting, facilitating strain transfer between the southern Gulf of Suez and the northern Red Sea domains^22^. Its structural configuration enhances fracture connectivity and plays a critical role in controlling focused groundwater recharge and subsurface flow.

## Hydrogeology

Hydrogeological information for the study area was compiled from previous investigations and available well data. Most groundwater wells in the region yield saline to brackish water, reflecting limited recharge and strong evaporation effects in this arid environment^24,25^ (Figs. 1b and 2). Quantifying this limited recharge^26^, reported that during the November 1996 rainfall event, only approximately 39.502 million m³ (14.38% of total precipitation) infiltrated as recharge, while 222.008 million m³ became surface runoff. This highlights that the dominant hydrologic process in the region is the dominance of rapid overland flow relative to infiltration. Accordingly, recognizing potential zones where this flow is captured, such as fractures, watersheds, channels, and other structurally controlled pathways, becomes important for groundwater management and for groundwater potentiality mapping. Based on the hydrogeological setting and well distribution, three main aquifer systems characterize the region^26^ (Supplementary Table S1 online):

1. **The fractured basement aquifer system**: It is the main source of fresh groundwater in the study area. The primary recharge zones are located in the western part of the area, where igneous and metamorphic rocks are exposed. These formations receive recharge primarily from direct precipitation and occasional torrential floods. The basement aquifer consists of two main water-bearing formations: granitic and metavolcanic, with groundwater occurrence controlled entirely by secondary fracture porosity.
2. **The Middle Miocene carbonate aquifer system**: It is primarily composed of reefal limestone, with inter-bedded layers of evaporites, clay, and shale. Groundwater productivity at G. Abu Sha’ar El-Qibli is relatively low, with recharge occurring through precipitation that infiltrates downward via fractures and joints during heavy rainfall storms, but is often limited by the overlying low-permeability layers.
3. **The Quaternary aquifer system**: It primarily consists of main channels and downstream areas of major wadis. The Quaternary aquifer is primarily composed of silt, poorly sorted sand, gravel, and rock fragments originating from the surrounding country rocks, with a thickness range of 5 to 20 m at Wadi Bali. The aquifer recharge is based on the infiltration from rainfall and runoff water during the flash flood.

The Red Sea Hills, built of Precambrian igneous and metamorphic rocks, form the dominant recharge area for the fractured basement aquifer due to their high fracture density. Foot slope hills composed of sedimentary rocks, such as Gabal Abu Sha’ar El-Qibli, Dishet El-Dabaa, and the surrounding coastal escarpments near Hurghada, act as secondary recharge zones for both the Middle Miocene carbonate aquifer and the Quaternary wadi deposits^27^. Overall, groundwater occurrence in the study area is highly dependent on structural pathways, fracture networks, and wadi-channel dynamics, emphasizing the need for integrated geospatial and geophysical techniques to delineate favorable groundwater zones.

## Geospatial data and methodology

### Geospatial thematic layers preparation

**Fig. 3:**
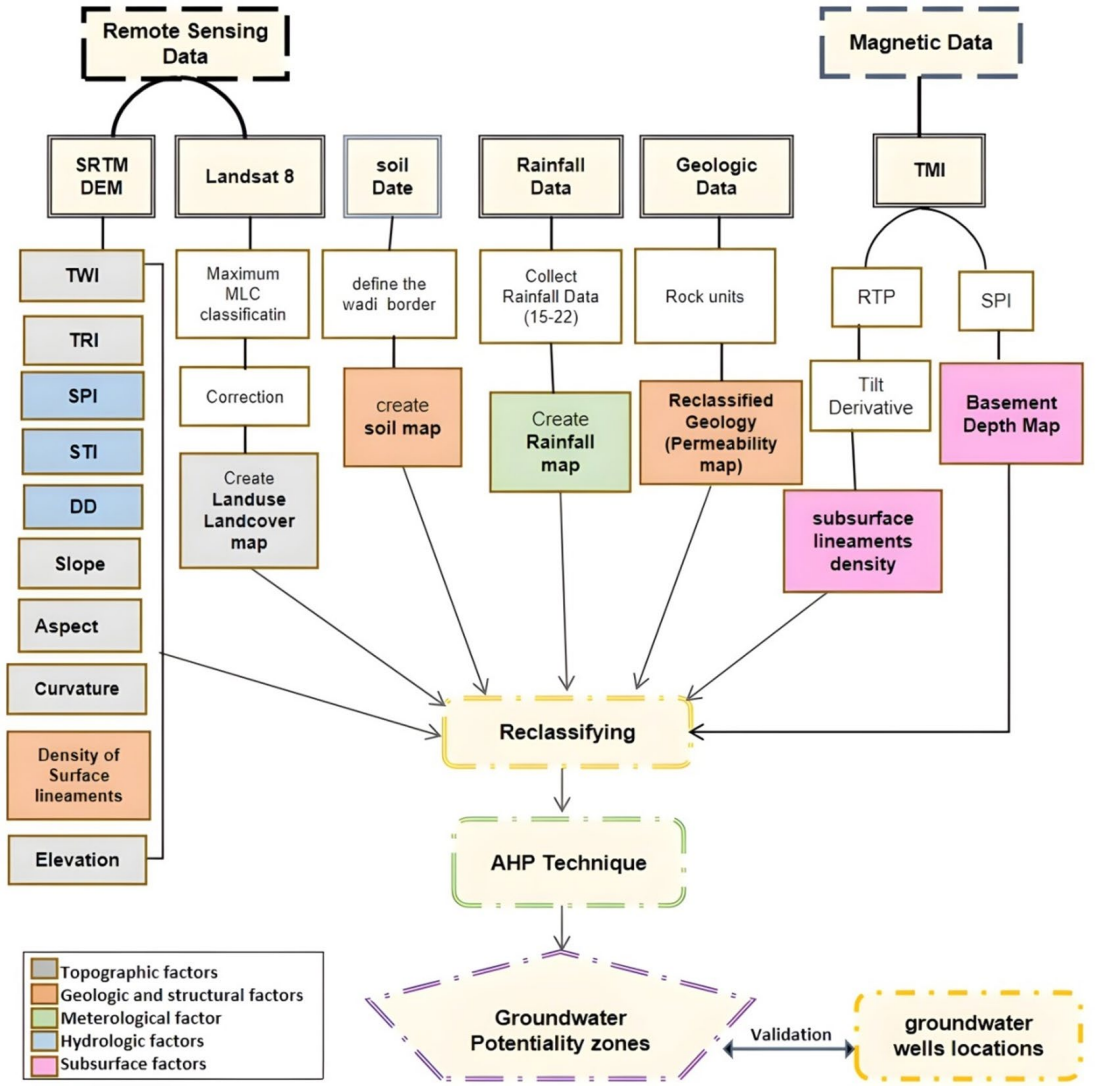
Flowchart shows the sixteen thematic layers and their source of data that utilized in the current study. (Generated by ArcGIS v.10.8. https://www.esri.com/ en- us/arcgis/).

In the current study, remote sensing (RS) was integrated with geophysical data through geographic information systems (GIS) techniques to develop a potential groundwater map in the study area for analyzing water paths and make a reliable model of groundwater potential zones in arid regions. The combined application of these multiple data enhances the understanding of both surface and subsurface controls on water movement, contributing to a more accurate delineation of groundwater potential zones. Figure 3 indicates the flowchart followed in the study that highlights the utilized data and methodology.

A total of sixteen thematic layers controlling groundwater occurrence were compiled and analyzed (Table 1). These layers are widely applied in groundwater potential studies (e.g^14,28–34^..,. They represent the key characteristics of the study area that influence the movement and storage of water. The layers were grouped into five categories: (i) Topographic; (ii) Geologic and structural; (iii) Meteorological; (iv) Hydrological; and (v) Subsurface factors (Table 1). The topographical LULC factor was classified using a Landsat 8 satellite image into four classes: barren land, barren rocky land, vegetation area, and buildup area (Table 2). Landsat imagery is widely used in LULC studies because of its suitable spatial resolution, multispectral capabilities, and long-term data availability, which provide consistent and reliable information on surface features^35^. The Maximum Likelihood Classification (MLC) method was performed to attain the LULC layer. The MLC is a supervised classification technique that assigns pixels to the class with the highest probability based on statistical parameters, making it one of the most widely used approaches for LULC mapping^36^. The results were subsequently refined and corrected to enhance accuracy.

**Table 1:** The geospatial and geophysical datasets used in the current study for groundwater potential assessment.

Category	Data source	Spatial resolution/scale	Thematic layer	Hydrogeological significance
**Topographic**	Shuttle Radar Topography Mission (SRTM DEM) downloaded from USGS (https://earthexplorer.usgs.gov/)	30 m	Elevation	Controls runoff, infiltration, and recharge
			Slope	
			Aspect	
			Curvature	
			Topographic roughness index (TRI) TRI= (Σ(Zc − Zi)^2^)^2^ where Zc is the central pixel elevation, and Zi is the neighboring pixel elevation^34^	
			Topographic wetness index (TWI) TWI = ln(flow accumulation/tan(slope))^32^	
	Landsat 8 downloaded from USGS (https://earthexplorer.usgs.gov/)		Land-use/Land-cover (LULC)	Reflects land cover impact on recharge (buildup areas: impervious surfaces, while barren lands: better water infiltration)
**Geologic and structural**	Geological map^23^	1:500,000	Geology (permeability)	Governs porosity and permeability
	FAO Soils Portal downloaded from the FAO Soils Portal (Food and Agriculture Organization, https://www.fao.org/soils-portal/en/)	~ 1 km	Soil type	Influences infiltration capacity
	SRTM DEM	30 m	Density of surface lineaments	Indicates fracture-controlled flow paths
**Meteorological**	CHRS Data Portal (https://chrsdata.eng.uci.edu/)	0.04°	Rainfall	Primary recharge source
**Hydrological**	SRTM DEM	30 m	Drainage Density (DD)	Runoff–infiltration balance
			Stream power index (SPI) SPI= flow accumulation * tan (slope)^30^	Flow energy and sediment transport
			Sediment transport index (STI) STI= (flow accumulation/22.13)^0.6^ * (sin (slope)/0.0896)^1.3^^30^	
**Subsurface**	Aeromagnetic data (Tilt angle derivative; TAD)	1:50,000	Subsurface lineaments (subsurflin)	Buried structural pathways
	Aeromagnetic data (Source parameter imaging; SPI)		Basement depth (SPImag)	Sediment thickness and storage potential

The geology thematic map was categorized into four categories of rocks based on their porosity and permeability. According to^4,7,37^, these categories were, Group A: with very high porosity like the Wadi deposits; Group B: with high porosity as Dakhla fm.; Group C: with intermediate porosity like Meta-sediment; and Group D: with low porosity like pink Granite. Regarding the coarse resolution of soil data, the classified soil map was refined along Wadi boundaries using the Landsat 8 satellite image. The surface lineaments were extracted from multi-azimuth hill-shade images derived from the SRTM DEM using PCI Geomatica software (developed by the Canadian company PCI Geomatics, CATALYST Earth; https://catalyst.earth/customer-center/tutorials/). The extracted lineaments were merged to get the highest resolution, various surface linear features possibilities and trends, as well as converted into a density map to identify structurally favorable zones for groundwater movement.

Rainfall data for the period 2015–2022 were processed to generate an annual precipitation layer, representing the primary source of groundwater recharge in the study area. The Hydrological DD layer was derived using the Strahler stream ordering method^38^. Regarding the subsurface factors, the aeromagnetic dataset used in this study was acquired by the Aero-Service Division of the Western Geophysical Company of America in 1984^39^. The survey was conducted along NE–SW-oriented flight lines spaced at approximately 1.5 km, with NW–SE tie lines at 10 km intervals. This survey configuration ensured adequate spatial coverage for detecting both regional and local magnetic anomalies associated with subsurface geological structures.

To reduce the effects of the Earth’s magnetic field inclination and declination, the total magnetic intensity (TMI) data were transformed using Reduction to the Pole (RTP)^40,41^, which repositions anomalies directly over their causative sources and facilitates more accurate structural interpretation. Following RTP transformation, derivative-based filtering techniques were applied to enhance structural features. In particular, the TAD was used to delineate subsurface structural boundaries and fault systems. The tilt angle (T) is defined from the first derivatives of the magnetic field in the horizontal and vertical directions. The TAD method enhances both strong and weak anomalies and is particularly effective in identifying linear features such as faults and lithological contacts. Notably, the zero-contour lines of the TAD map correspond to the edges of magnetic sources, marking zones of contrasting physical properties and structural discontinuities^42^. These features were interpreted as subsurface lineaments which representing potential preferential pathways for groundwater movement.

Additionally, the SPI technique was applied to estimate the depth to magnetic sources^43^. This method is based on the local wave number of the magnetic field, which relates directly to the depth of the causative source. The local wave number is computed using the first and second derivatives of the magnetic field and the depth to the magnetic source is then estimated from the inverse of the maximum local wave number^44^. The SPI method assumes simplified source geometries, typically vertical or near-vertical contacts and uniform magnetization. Although the SPI-derived depths are subject to uncertainty (sensitivity to noise), its suitability for rapid and continuous depth estimation of complex geological settings makes it commonly used in many studies^45,46^. Therefore, in this study, the derived basement depth layer (SPImag) is interpreted as an approximate indicator of sediment thickness and structural configuration rather than an exact measure of depth. The integration of these subsurface constraints significantly strengthens the reliability of the groundwater potential model. The processing and analysis methods of the magnetic data were performed by Oasis montaj v.8.4 software (https://my.seequent.com/). Additionally, the final factors maps were generated and classified using the different spatial tools (e.g., Line Density tool and Jenks natural breaks classification method) that that presented in ArcGIS 10.8 software (https://www.esri.com/ en- us/arcgis/).

### AHP method, validation and sensitivity analysis

The AHP method is a knowledge-driven multi-criteria decision-making method that integrates quantitative and qualitative factors using expert-based weighting and theoretical understanding^47,48^. It provides a transparent and systematic framework for evaluating complex problems. AHP was used to evaluate how different thematic layers and their attributes affect groundwater potential. It works by comparing the factors in pairs and assigning weights to each one, creating a priority scale that shows their relative importance in determining groundwater potential^49^. The AHP method is essentially based on expert judgment to determine criteria weights through pairwise comparisons. While this study employed a structured judgment matrix validated for consistency (CR < 0.1) and grounded in the site-specific hydrogeological understanding, it is important to acknowledge that this represents one possible weighting scenario. Alternatively, weighting schemes such as the entropy method, frequency ratio, or machine learning models could produce different weight distributions.

In the current study, the pairwise comparison matrix and the normalized matrix were performed on the resultant sixteen thematic layers to compare their importance, and generate consistency index (CI), as well as the consistency ratio (CR)^50^;. The pairwise comparison matrix of the utilized layers to build the AHP model can be found in Supplementary Table S2 online. According to^47,51^, a model is considered consistent when the CR is below 0.1. If the value is higher, the priorities must be revised. CI shows how much inconsistency exists and is calculated using the following formula:

1 $$\:\mathrm{C}\mathrm{I}=\frac{{\uplambda\:}\mathrm{m}\mathrm{a}\mathrm{x}-\mathrm{n}}{\mathrm{n}-1}=\frac{16.118-16}{15}=\:0.008$$

where (λmax) is the greatest eigenvalue, and the variable (n) is the number of thematic layers. While CR, according to^40^ developed as follows:

2 $$\:\mathrm{C}\mathrm{R}=\frac{\mathrm{C}\mathrm{I}}{\mathrm{R}\mathrm{I}}=\frac{0.008}{1.6}=\:0.0049$$

where RI is the random CI (RCI). Since the CR value is acceptable, the weight% of each layer was approved as shown in Tables 2 and 3. The validation of the AHP model was conducted using groundwater well data compiled from previous studies^24–26^, comprising a total of 57 wells. To ensure independent model evaluation, the dataset was randomly divided into training (60%) and validation (40%) subsets. The validation wells were spatially overlaid on the groundwater potential map to evaluate their agreement with predicted high-potential zones. In addition, randomly generated non-well points were used to represent absence conditions. The continuous groundwater potential index was reclassified into a binary map using a threshold corresponding to the high-potential class, which reflects the most favorable hydrogeological conditions for groundwater occurrence. Model performance was assessed using the Receiver Operating Characteristic (ROC) curve and the Area Under the Curve (AUC). A confusion matrix was also constructed to compute sensitivity and specificity, providing additional measures of predictive accuracy^52^.

Sensitivity analysis is commonly used to evaluate the influence of input parameters on model results and to examine the robustness of the selected layers/factors. In the present study, a single-parameter sensitivity analysis was conducted to assess the relative contribution of the utilized factors used in groundwater potentiality mapping. This approach quantifies the actual influence of each factor on the final model results. The effective weight (eW_i_) for each factor was calculated using the corresponding theoretical weight (thW_i_) and rating value (R_i_), according to the following expression^53^:

3 $$\:\mathrm{e}\mathrm{W}\mathrm{i}=\frac{\mathrm{t}\mathrm{h}\mathrm{W}\mathrm{i}\mathrm{*}\:\mathrm{R}\mathrm{i}\:}{\mathrm{V}\mathrm{I}\mathrm{i}}*100$$

where, VI_i_ is the vulnerability index (sum of the products of all factors).

**Table 2:** The utilized sixteen layers in the current study with their importance, ranks, and weights.

Layer name	Rule/Relation	Rank	Weight	Weight%
**Density of Surface** **lineament**	Direct^54,55^	5- 0.0013:0.0022 4 − 0.00097:0.0013 3 − 0.00068:0.00097 2 − 0.00036:0.00068 1- 0:0.00036	0.125	13
**Geology (permeability)**	Direct^56^	5- Group A 4- Group B 3- Group C 1- Group D	0.127	13
**Slope**	Inverse^57^	5 − 0:5.6 4–5.6:13.4 3–13.4:23 2 − 23:34 1 − 34:76	0.116	12
**Rainfall**	Direct (primary source of surface water)	5–18:22 4- 16–18 3- 14:16 2 − 12:14 1- 0:12	0.11	11
**subsurflin**	Direct	5- 0.46:0.8 4 − 0.33:0.46 3 − 0.22:0.33 2 − 0.085:0.22 1- 0:0.085	0.069	7
**SPImag**	Direct	5- 2970:5230 4 − 1966:2970 3 − 1271:1966 2- 730:1271 1- 300:730	0.069	7
**DD**	Inverse^58^	5 − 0:2.5 4–2.5:3.5 3–3.5:4.4 2–4.4:5.4 1–5.4:7.45	0.061	6
**LULC**	LULC provides useful information about infiltration, soil moisture, and how much the region depends on groundwater^59^.	5- Barren Land 4- Vegetation area 2- Build up area 1- Barren rocky land	0.061	6
**Soil**	Direct (coarser soil)^59^	5- sandy loam 2- loam	0.042	4
**TWI**	Direct^60^	5- 1.76:12.46 4- (−1.74):1.76 3- (−4.05):(−1.74) 2- (−5.76):(−4.05) 1- (−9.36):(−5.76)	0.042	4
**Elevation**	Inverse^61^	5–10:200 4 − 200:500 3- (−9):10 2–500:750 1 − 750:1940	0.04	4
**Aspect**	Variable depending on the targeted location and the direction of slope faces^62,63^	5- flat 4-northwest &southeast 3-northeast & east 2- north & south 1- west& south west	0.04	4

**Table 3:** Continued.

Layer name	Rule/Relation	Rank	Weight	Weight%
**Curvature**	Inverse^64^	5- flat 3- concave 2- convex	0.037	3
**SPI**	Inverse^65^	5 − 0:(−6.9) 4- (−6.9):(−2.5) 3- (−2.5):(−0.48) 2- (−0.48):2.39 1–2.39:12.34	0.021	2
**TRI**	Inverse^66^	5 − 0:0.345 4 − 0.345:0.437 3 − 0.437:0.52 2 − 0.52:0.62 1 − 0.62:0.9	0.021	2
**STI**	Inverse^67^	5 − 1:431 4 − 431:1848 3 − 1848:4374 2 − 4374:8750 1 − 8750:15712	0.02	2

## Results

### Analysis of geospatial thematic layers

#### Topographic layers

**Fig. 4:**
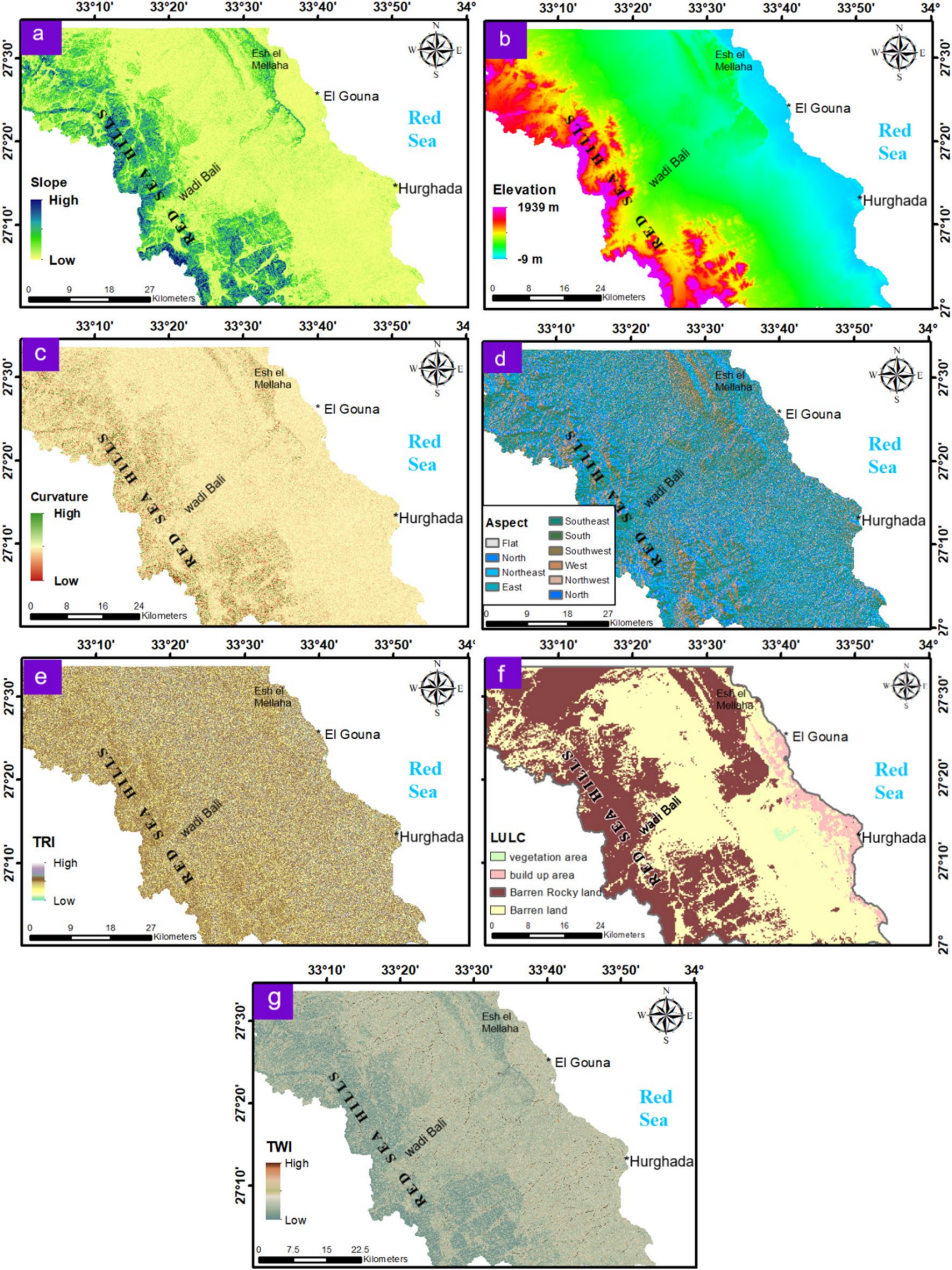
Maps of the topographic influencing factors of the study area: (**a**) Slope; (**b**) Elevation; (**c**) Curvature; (**d**) Aspect; (**e**) TRI; (**f**) LULC; (**g**) TWI. These maps control the runoff and infiltration, as well as their effect on recharge. (Generated by ArcGIS v.10.8. https://www.esri.com/ en- us/arcgis/).

Regarding the importance of these layers in surface water movement and groundwater recharging (Table 1), it was necessary to map and analyze many of them in the current study. Elevation and slope show a clear spatial relationship, where high elevated areas (~ 1,939 m) in the western Red Sea Hills and northeastern G. Esh El Mellaha correspond to steep slopes (up to 75.8°), which favor rapid runoff and limited infiltration (Fig. 4a and b). In contrast, most of the study area is characterized by low slopes and gradually decreasing elevation toward the Red Sea coast, reaching − 9 m, which promotes surface water retention and infiltration. Figure 4 (c - f) indicates that the barren rocky lands exhibit abrupt curvature and high TRI values that reflect terrain irregularity, sharp discontinuities, and limited infiltration potential. The moderately elevated areas show flat curvature, with variable roughness index values. Aspect analysis reveals dominant north to east facing slopes, which direct surface runoff toward lowland areas and permeable barren lands (Fig. 4d and f).

The LULC map indicates that the buildup class is concentrated along the Red Sea coast, particularly in the cities of El Gouna and Hurghada (Fig. 4f). It also shows that the preferred barren land class extends in the northwest–southeast trend (Tarboul and West Hurghada basins) and along the wadies, with scattered vegetated areas (Figs. 2 and 4f). Unlike barren rocky land, these barren surfaces allow higher infiltration relative to runoff. The TWI map highlights strong spatial contrasts in moisture accumulation (Fig. 4g). Low TWI values dominate the mountainous zones, reflecting steep and well-drained terrain. In contrast, moderate to high TWI values occur in the flat plains and main wadis, indicating areas of potential soil moisture accumulation and groundwater recharge.

Based on the analysis of these layers, the geomorphological favorable zones for groundwater recharge are relatively delineated through the NW-SE trending flat barren lands with north-, northeast-, and east-facing slopes that preserve more moisture.

#### Geologic and structure layers

**Fig. 5:**
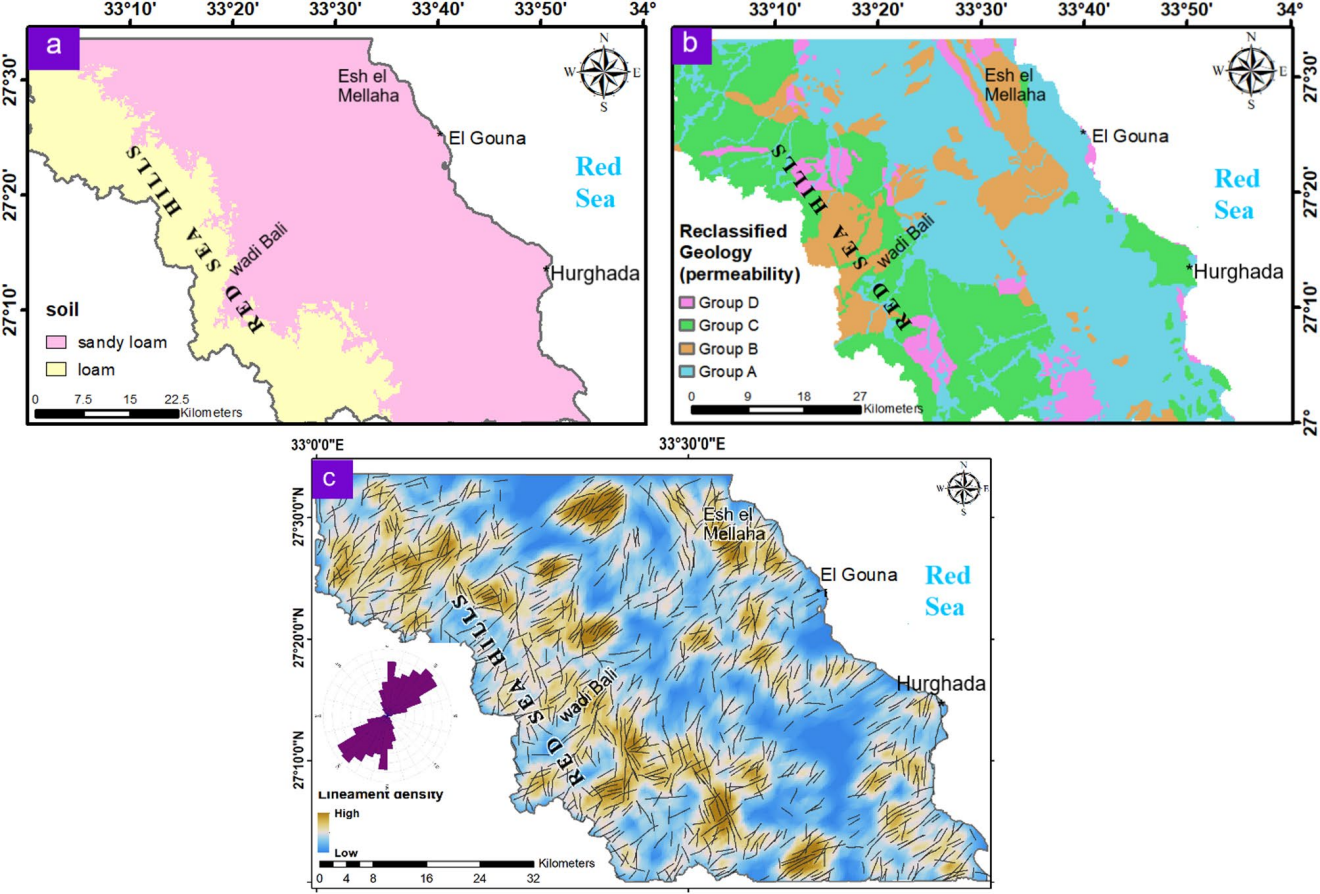
Maps of the geologic and structure influencing factors of the study area: (**a**) Soil; (**b**) Geology; (**c**) Density of surface lineaments. These maps indicate areas control the surface water flow paths and influence the infiltration capacity. (Generated by ArcGIS v.10.8. https://www.esri.com/ en- us/arcgis/).

Geological formations, soil properties, and structural features collectively exert strong control on groundwater movement and storage. In the investigated area, sandy loam dominates most of the region, extending from the western highlands toward the coast along a NW–SE trend. This soil type is more favorable for recharge because its infiltration capacity (10–20 mm/h) is higher than that of loam (5–10 mm/h) which is restricted to the western part (Fig. 5a). The apparent uniformity of soil thickness is influenced by the original image resolution as well as environmental and tectonic controls, making it unreliable on its own. Therefore, geological and structural lineaments were incorporated to improve the accuracy of groundwater assessment.

The geological units were grouped into four permeability classes (Table 2; Fig. 5b). Groups A and B, characterized by high porosity and permeability, are widely distributed and coincide with vegetated LULC patches, indicating enhanced water availability and subsurface storage. These units also follow a NW–SE spatial pattern consistent with regional structural trends (Fig. 4). The exposed geological units are affected by the tectonic origin of the area as indicated by the extensive distribution of the extracted surface lineaments (Fig. 5c). The high elevated lands in the west and northeast are characterized by the high density of the lineament, particularly at the boundaries of the units. The dominant trend orientation of these lineaments follows NEE-SWW and NNE-SSW trends with minor NNW-SSE one, as illustrated in the rose diagram (Fig. 5c). This layer guides surface runoff and represents preferential pathways for infiltration and groundwater movement.

#### Meteorological and hydrologic layers

**Fig. 6:**
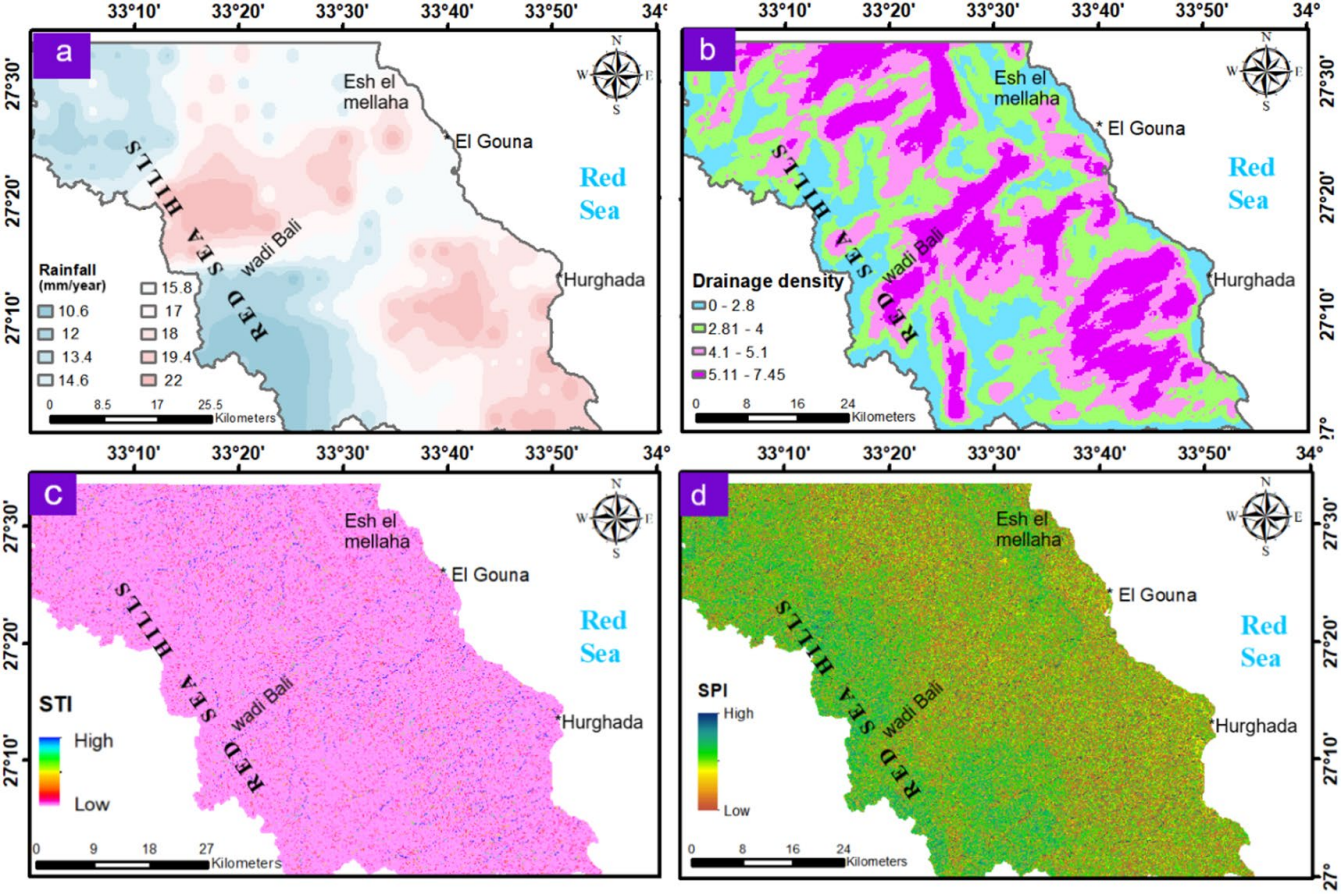
Maps of the meteorological and hydrologic influencing factors of the study area: (**a**) Rainfall rate; (**b**) DD; (**c**) STI; (**d**) SPI. These maps display the primary groundwater recharge source, surface flow energy and sediment transport. (Generated by ArcGIS v.10.8. https://www.esri.com/ en- us/arcgis/).

These layers collectively define surface water availability and its potential contribution to groundwater recharge. In the study area, the highest rainfall rate equals 22 mm/year occurring in the western mountainous areas and near the Hurghada–El Gouna coastal zone (Fig. 6a). Consequently, groundwater recharge is largely dependent on short-duration intense rainfall events and efficient infiltration through favorable geomorphic and structural settings. The DD map reflects the combined influence of rainfall, lithology, and structure (Fig. 6b). This map indicates structurally controlled surface with runoff dominates and decreases the potentiality of infiltration, thereby diminishing the influence of rainfall on groundwater recharge in these areas (Fig. 6b). This is considered one of the reasons that lead to the frequent occurrence of flood risks affecting the region, and it increases the challenges facing those interested in exploring groundwater, thus increasing the value of the current study.

Regarding the importance of STI and SPI factors (Table 1), high STI and SPI values are related to the steep catchments or main drainages/streams (e.g., Wadi Bali) that indicate strong erosive power and sediment transport (Fig. 6c and d). Low values occur in coastal plains, wadis, and alluvial fans, where reduced flow energy favors sediment deposition and potential infiltration. The major SPI values distribution also effectively visualizes the tectonic architecture in the study region. All the results of the meteorological and hydrologic layers suggest that the groundwater recharge typically occurs in wadi bottoms, alluvial fans, or structural depressions filled with reduced erosive power streams and coarse sediments.

#### Subsurface layers

**Fig. 7:**
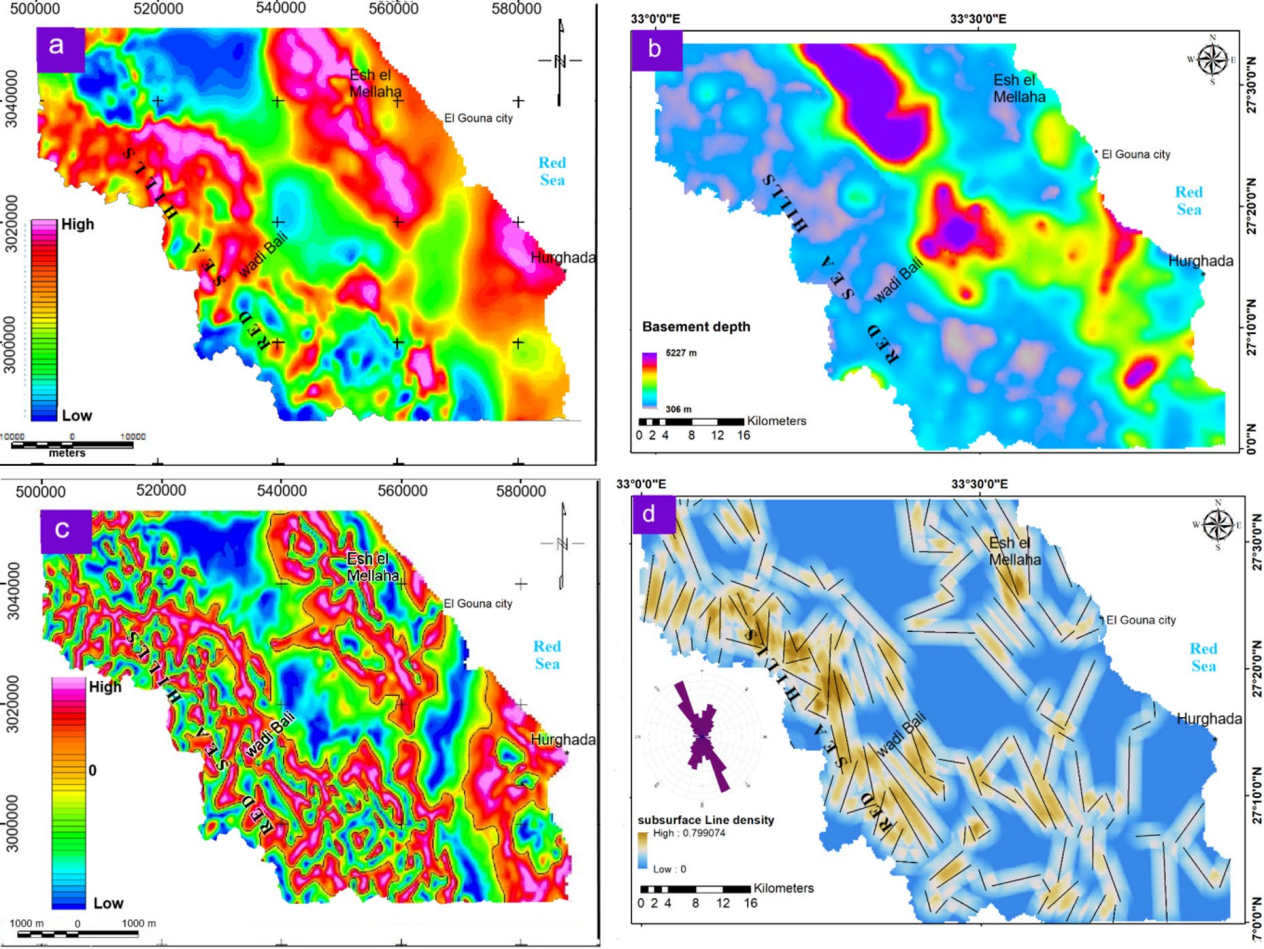
Maps of the subsurface influencing factors derived from aeromagnetic analysis: (**a**) SPImag; (**b**) Basement Depth; (**c**) TAD; (**d**) Density of subsurface faults. The resultant basement depth and density of subsurface faults maps reveal the subsurface sediment thickness and structural pathways, respectively. (Generated by ArcGIS v.10.8. https://www.esri.com/ en- us/arcgis/).

Based on the types of aquifers in the study area and the previous groundwater exploration studies (Sects. 2–4; Table 1), subsurface layers provide critical constraints on groundwater storage and flow by revealing sediment thickness and structural architecture. The SPImag results delineate depth to magnetic basement, ranging from shallow (~ 300 m) to very deep (~ 5220 m) sources (Fig. 7a and b). The results indicate zones of increased sediment thickness, particularly within the central NW–SE trending sector and coastal structural basins (NE–SW trend). However, sediment thickness is not considered a direct indicator of groundwater storage, as occurrence depends mainly on lithology, permeability, and structural connectivity, and is enhanced only where favorable conditions coexist. Shallow basement areas (shallow magnetic sources) correspond to exposed or near surface crystalline rocks in the west and northeast (Red Sea Hills and G. Esh El Mellaha), consistent with mapped geological units and existing well data (Supplementary Table S3 online).

The subsurface lineaments layer was generated from the TAD map (Fig. 7c) that exhibits the linear trends accompanied by the structural framework. The subsurface lineaments density map highlights major fault systems that control groundwater movement. High fault density in uplifted areas enhances secondary porosity and facilitates recharge, whereas low-density zones delineate structural basins bounded by NW- and NE-trending faults (Fig. 7d). The trend analysis of these faults shows two main predominant directions, including the NNW-SSE and NNE-SSW ones. These trends match the regional tectonic patterns that confirm the structural control on groundwater flow. The interaction between fault-controlled recharge, lithological properties, and basin geometry contributes to the development of favorable groundwater potential zones, rather than any single factor acting independently. Table 4 summarizes the characteristics of the layer maps or their dominant spatial trends that potentially influence groundwater in the area.

### AHP model for groundwater potentiality

**Fig. 8:**
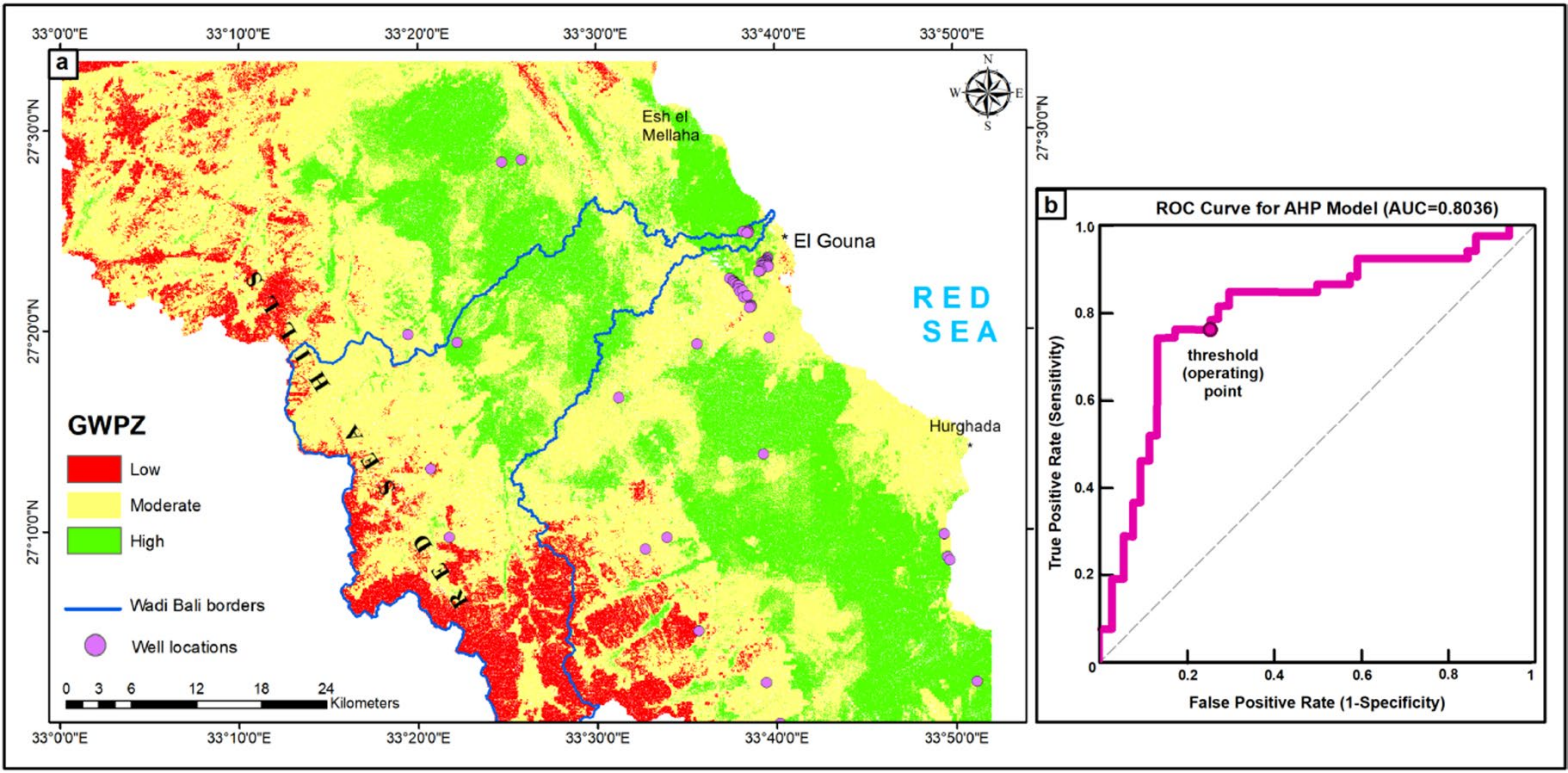
**(a)** The AHP-based GWPZ model shows high groundwater potential concentrated in Wadi Bali, coastal plains, and major structural depressions; **(b)** ROC curve for the AHP model illustrating the trade-off between sensitivity and false positive rate. The optimal threshold maximizes sensitivity-specificity balance, with good model performance. (Generated by ArcGIS v.10.8. https://www.esri.com/ en- us/arcgis/).

According to the previous hydrological studies, it is evident that the groundwater obtainability is not uniform in space and time. Consequently, a better understanding of the groundwater potential in arid coastal areas is an important trend towards good planning and sustainable development in these areas. AHP is a valuable approach that analyzes the various influencing layers and delineates accurate groundwater potential zones^4,9^. The groundwater potential map derived from the AHP model was classified into three categories of potentiality: high, moderate, and low, with spatial extension 36.5%, 54%, and 9.5% respectively (Fig. 8a). The potentiality map demonstrates that areas characterized by localized low-lying barren lands, deep depressions and coastal plains exhibit high groundwater potential, while hard rock mountainous regions with steep slopes are associated with low groundwater potentiality. The moderate potential zones are the most widespread in the region, where they generally occur in the valleys and areas of moderate to high drainage density. Regarding the Wadi Bali borders, the high potentiality zones are concentrated at the central part and the mouth of the Wadi with an aerial spread of 112,128 Km^2^ (25% of the wadi area) (Fig. 8a). The zones’ distribution is mainly related to the structural features and depth of the basement.

**Table 4:** Summary of the main spatial trends for the utilized thematic layers.

Category	Thematic layer	Dominant spatial pattern
**Topographic**	Elevation	High relief in western (Red Sea Hills) and NE mountains (G. Esh El Mellaha); lowlands toward the coast
	Slope	Steep in Red Sea Hills and G. Esh El Mellaha; gentle in central and coastal areas
	Aspect	Dominantly N, NE, and E facing
	Curvature	Abrupt in rocky terrains; flat in plains and wadis
	TRI	High in rugged mountains; low in plains
	LULC	Barren land dominant in NW-SE plains (structural basins) and wadis; built-up along coast
	TWI	High along wadis and lowlands; low in mountains
**Geologic & Structural**	Geology	High permeability units (wadi deposits, Dakhla Fm.) dominate NW–SE zones
	Soil	Sandy loam widespread; loam in western areas
	Density of surface lineament	High in western and NE highlands as well as along unit boundaries
**Meteorological**	Rainfall	Slightly higher in western mountains and coastal zones
**Hydrologic**	DD	High in mountainous and structurally controlled areas; low in plains
	STI	High in steep channels; low in plains and fans
	SPI	High in mountains and confined channels; low in lowlands
**Subsurface**	Basement depth	Deep basins in central NW-SE belt and coastal depressions
	Density of subsurface lineament	High along basement highs; low in basins

In the present study, the delineated high potential zones were cross-validated with the known well data that found in Supplementary Table S3 online. This is an important step in assessing the model’s accuracy due to the diversity of well data from different groundwater levels and types of aquifers. The validation accuracy (the AUC value) of the modeled high potential zones reaches 80.3% (Fig. 8b), which indicates strong model performance^67^. Based on the selected threshold, the model achieved a sensitivity of 0.74 and a specificity of 0.74, reflecting a balanced ability to correctly identify both groundwater occurrence and non-occurrence locations. These results confirm the reliability of the groundwater potential map at the regional scale. Consequently, it demonstrates that structural features, lithology, and slope are the dominant controls on groundwater recharge and storage, emphasizing the structurally controlled nature of the aquifer system.

**Fig. 9:**
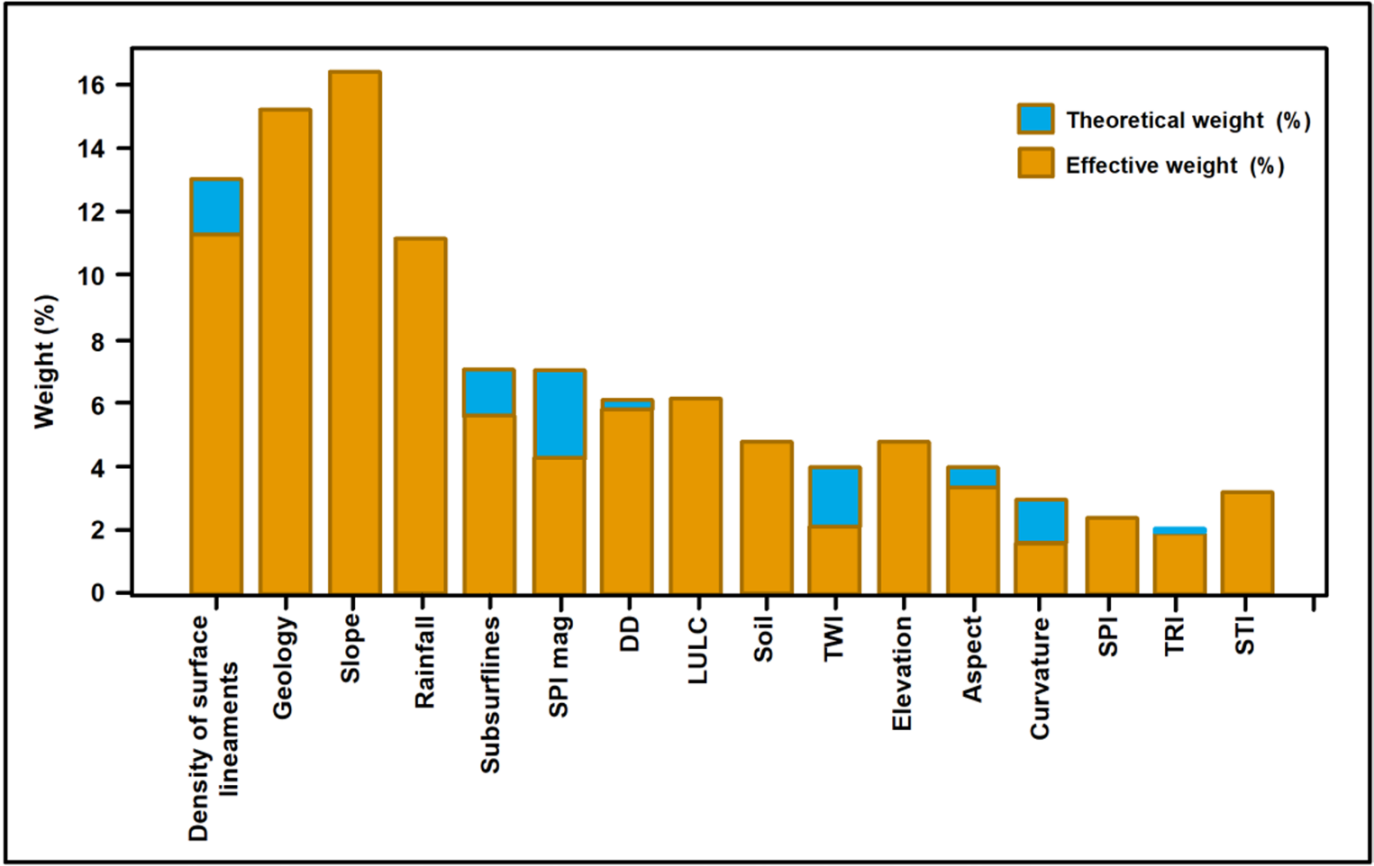
Sensitivity analysis results calculated from the single parameter analysis showing the relative influence of thematic factors on groundwater potential mapping. (Generated by ArcGIS v.10.8. https://www.esri.com/ en- us/arcgis/).

To further evaluate the relative contribution of the factors used in the model, a single parameter sensitivity analysis was performed. This analysis compares the assigned theoretical weights with the effective weights derived from the spatial modeling results in order to assess the influence of each layer/factor on the final groundwater potential index. The results (Table 5; Fig. 9) show that factors related to geology (permeability), surface structural lineaments, and topographic conditions (slope) show relatively high effective weights, emphasizing their importance in regulating infiltration pathways and groundwater storage. Conversely, several secondary layers including curvature and TRI exhibit comparatively lower effective contributions, suggesting a more localized or indirect influence on groundwater accumulation. Overall, the close correspondence between theoretical and effective weights (differences < 5%) confirms the robustness of the AHP weighting scheme and indicates that the model successfully captures the hydrogeological processes governing groundwater distribution within the structurally complex rift setting of the southern Esh El Mellaha region.

**Fig. 10:**
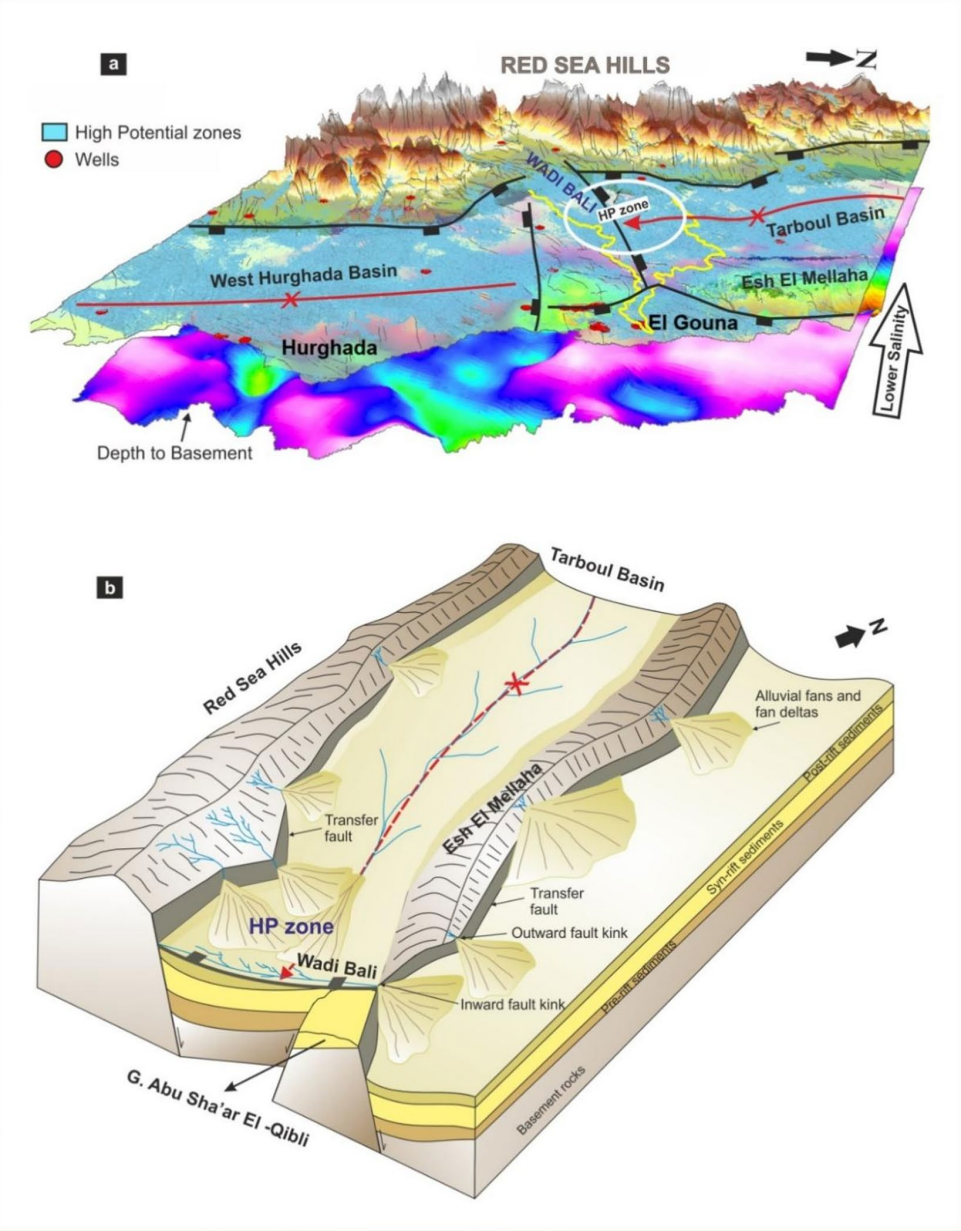
(**a**) 3D perspective visualization integrating DEM, groundwater potential zones, basement depth, structural framework, and well locations, illustrating spatial relationships among key controlling factors, as well as the high priority zone (HP zone) for drilling. (**b**) Conceptual model summarizing the interpreted structural controls on groundwater accumulation, including fault-related folding, hard-linked faults, and the Bali accommodation zone. The model is interpretative and not quantitatively constrained. (Generated by ArcGIS v.10.8. https://www.esri.com/ en- us/arcgis/, and CorelDraw X5, https://www.coreldraw.com).

To provide a detailed visualization of the hydrogeological system and groundwater potentiality, a representative three-dimensional (3D) model was developed integrating well locations, AHP-derived high-potential zones, and the most influential surface and subsurface layers (Fig. 10a). The model highlights priority groundwater targets in the northern-central part of the study area, situated between uplifted basement blocks of the Red Sea Hills and the southern sector of G. Esh El Mellaha. These zones are characterized by relatively thick sedimentary cover, high densities of surface and subsurface lineaments, and moderate groundwater levels with comparatively low salinity. Such conditions indicate favorable hydrogeological settings for groundwater accumulation and extraction. In contrast, other zones, although showing moderate-to-high potential, are considered less suitable due to their proximity to high-salinity wells. The model therefore serves as a practical decision-support tool for identifying optimal drilling locations, improving exploration efficiency, and supporting sustainable groundwater resource management in arid environments.

**Table 5:** The sensitivity results of the single parameter analysis (ranging from higher to lower effective weights).

Thematic layer	Theoretical weight%	Effective weight%
**Slope**	12	16.4
**Geology (permeability)**	13	15.2
**Density of Surface** **lineament**	13	11.3
**Rainfall**	11	11.2
**LULC**	6	6.2
**DD**	6	5.8
**subsurflin**	7	5.6
**Elevation**	4	4.8
**Soil**	4	4.8
**SPImag**	7	4.2
**Aspect**	4	3.4
**STI**	2	3.2
**SPI**	2	2.4
**TWI**	4	2.1
**TRI**	2	1.9
**Curvature**	3	1.6

The AHP model accounted for the topographic complexity of the study area along with the subsurface geological characteristics that influence groundwater dynamics. This highlights the importance of integrating subsurface information with surface-derived parameters to achieve an overall understanding and accurate assessment of groundwater flow and potential zones^9^. Consequently, the integration of these datasets enhances the reliability of groundwater potential mapping and provides a robust framework for sustainable groundwater resource management in the region.

Despite the robustness of the integrated AHP framework, several limitations should be considered:

- Spatial resolution mismatch among input datasets (e.g., high-resolution DEM versus coarser soil and rainfall data) required resampling to a common grid, which may introduce generalization effects and reduce local variability. This may oversimplify subsurface conditions and smooth spatial heterogeneity in key zones such as wadis and alluvial fans. While the model remains reliable at the regional scale (the area of study ~ 3504.7 km²), the precision of groundwater potential mapping may be reduced at finer, site-specific scales.
- Rainfall, DD, and erosive indices (SPI and STI) are highly sensitive to episodic storm events, which are difficult to generalize in extremely arid environments.
- Geophysical magnetic depth modeling and TAD-derived faults may not fully capture small-scale fractured zones that can strongly influence groundwater flow.
- Validation wells are unevenly distributed and represent different aquifer types, which introduces uncertainty into accuracy assessment.

These limitations indicate that higher-resolution datasets, expanded field verification, and additional geophysical surveys would further improve model reliability in structurally complex arid regions.

## Discussion

This study demonstrates that incorporating subsurface tectonic information into GIS-based groundwater potential modeling significantly improves the hydrogeological interpretation of groundwater distribution in structurally complex arid environments. The results demonstrate that groundwater distribution in the southern Esh El Mellaha region is strongly influenced by the regional tectonic architecture, with the assessed AHP factors largely reflecting surface expressions of deeper structural controls. A key contribution of this work is the integration of magnetic basement-depth modeling and subsurface fault mapping within a GIS-AHP framework, enabling groundwater potential to be evaluated in a tectonically complex rift-transfer zone. Two principal structural mechanisms exert primary control on groundwater occurrence: (1) fault-related folding and (2) hard-linked fault systems formed by interactions between rift-parallel faults and transfer structures. The 3D perspective view (Fig. 10a) provides a data-informed visualization of the spatial relationship between high groundwater potential zones, basement depth, structural framework, and well locations. It is intended to illustrate the consistency between independently derived datasets rather than to represent a fully quantitative subsurface model. In contrast, the conceptual model (Fig. 10b) synthesizes the genetic relationships among fault systems, sediment accumulation, and groundwater pathways. Accordingly, the 3D visualization should be interpreted as a qualitative integration of multiple datasets, while the conceptual model represents a simplified hydrogeological interpretation.

The Tarboul Basin provides a clear example of a fault-related folding controlling groundwater accumulation. The basin is expressed as a SE-plunging syncline developed along the downthrown side of the Red Sea Hills Bounding Fault. This geometry exerts a direct influence on hydrologic behavior. Surface runoff converges naturally toward the synclinal trough, while sediments derived from multiple wadis are funneled toward the basin axis (Fig. 6c). Thick Quaternary deposits preferentially accumulate within the plunge zone (Figs. 5b and 7b), creating favorable conditions for groundwater storage. In addition, subsurface flow is guided southward along the synclinal axis. The coincidence of high drainage convergence, thick sediments, and elevated AHP groundwater potential values explains the prominent high-potential zone identified in the southern Tarboul Basin. This relationship highlights the advantage of integrating subsurface structural interpretation with GIS-based multi-criteria analysis. It enables more reliable identification of tectonically controlled groundwater accumulation zones compared to approaches based solely on surface factors/indicators.

A second dominant control is associated with hard-linked faults that develop where N-S transfer faults intersect NW-SE rift-parallel normal faults. As described by^68^, inward fault kinks create localized depocenters where alluvial fans accumulate, whereas outward fault kinks promote sediment dispersal and broader drainage pathways. Both geometries are well developed along the Esh El Mellaha boundary fault, the Red Sea Hills bounding fault, and within the West Hurghada basin. In the El Gouna sector, the intersection of multiple linked faults forms an inward kink that favors fan deposition and enhances infiltration. These areas correspond to moderate-to-high AHP groundwater potential values and thick Quaternary cover (Supplementary Table S3 online), highlighting the quantitative relationship between fault linkage geometry and groundwater favorability. This is consistent with^21^ study, which concerned with analyzing the drainable porosity volumes for the upper aquifers of the alluvial fan, indicating low clay content and high coarse clastics. This condition is good for improving the reservoir properties.

The Bali Shear Zone represents a key ENE-trending structural corridor that transfers strain between the southern Gulf of Suez rift and the northern Red Sea. This zone is spatially aligned with the main course of Wadi Bali and is characterized by reactivated basement fabric that enhances both vertical and lateral permeability. During episodic flash-flood events, the shear zone acts as a preferential recharge pathway, facilitating infiltration and guiding groundwater movement toward the Tarboul and West Hurghada structural lows (Fig. 10b). This behavior illustrates the importance of transfer zones in linking surface hydrologic processes with subsurface groundwater systems in rifted terrains.

The strong spatial correspondence between magnetically inferred basement lows, drainage convergence, thick Quaternary sediments, and high AHP groundwater potential zones suggests that tectonic architecture and stratigraphic conditions jointly influence groundwater distribution (Fig. 10). However, groundwater occurrence in arid environments is controlled by the combined effects of structural, geomorphological, and climatic factors. In the study area, tectonic structures play a key role in creating an accommodation zone, and guiding subsurface flow pathways, while geomorphological conditions such as drainage density, slope, and sediment accumulation control surface runoff concentration and infiltration. Additionally, climatic variability, particularly the episodic nature of rainfall and flash-flood events, influences groundwater recharge processes. Therefore, the observed groundwater distribution reflects the interaction between tectonic controls, lithology, and surface processes rather than a single dominant factor. This integrated interpretation is consistent with the hydrogeological framework of the study area (Supplementary Table S1 online), where groundwater occurrence varies significantly between aquifer systems, as well as with the previous hydrogeological studies in arid regions^24^.

Although many ML approaches have recently been applied to groundwater potential mapping and often achieve high predictive accuracy, their performance strongly depends on the availability of large training datasets. In structurally complex and data-scarce regions such as the Eastern Desert, the GIS-AHP approach remains advantageous because it allows the integration of geological knowledge and structural interpretation within a transparent decision framework^69,70^. The consistency between the predicted high-potential zones and independent hydrogeological observations reported in previous studies^21,24^ further supports the reliability of the proposed approach. Additionally, the agreement between ROC-AUC results and confusion matrix metrics strengthens confidence in the robustness of the AHP-based approach under the given data constraints.

The correspondence between the sensitivity results and the mapped structural lows strengthens the interpretation that tectonically controlled depocenters and linked fault systems constitute the principal hydrogeological reservoirs in the region. The sensitivity results indicate that factors related to structural configuration and geological properties exert the strongest influence on the final groundwater potential index. In contrast, several purely geomorphometric variables contribute comparatively less. Overall, the sensitivity analysis not only confirms the robustness of the AHP weighting scheme but also demonstrates that the combined influence of tectonic architecture, geomorphological conditions, and climatic controls governs groundwater distribution within this rift-transfer zone. However, it is important to recognize that the relative importance of these factors is partly dependent on the AHP weighting scheme, which incorporates expert judgment despite satisfying the consistency ratio criterion (CR < 0.1). In this context, the higher weights assigned to geology and lineament density are supported by the hydrogeological framework of the study area, where groundwater occurrence varies significantly among aquifer systems, including fractured basement, Miocene carbonates, and Quaternary deposits, each characterized by distinct permeability structures and recharge mechanisms^26^.

Nevertheless, alternative weighting approaches (e.g., frequency ratio, entropy-based, ML methods) may yield different spatial distributions of groundwater potential by emphasizing statistical relationships rather than expert-driven criteria. Therefore, the presented results should be interpreted as a robust but method-dependent representation of groundwater favorability. Future studies integrating data-driven weighting schemes and additional hydrogeological constraints would further enhance model objectivity and predictive performance.

From a practical perspective, the results provide clear guidance for groundwater exploration and sustainable management. Priority drilling targets (HP zone) are identified in structurally controlled lows, particularly the southern Tarboul Basin west of Esh El Mellaha, where synclinal plunge geometry, linked fault-controlled alluvial fans, and thick sediment accumulation coincide. Transfer zones such as the Bali Shear Zone should be considered strategic recharge corridors and protected from excessive land-use modification. The integrated workflow demonstrates that a well-implemented AHP framework, combined with sensitivity analysis and geological expertise, provides a reliable and interpretable tool for groundwater potential assessment. It is applicable to other arid, tectonically complex rift settings, and it also helps reduce exploration risk and supports long-term water-resource planning.

## Conclusion

This study provides an integrated, multi-criteria evaluation of groundwater potential across the southern Esh El Mellaha region, revealing the dominant role of tectonic and structural controls in shaping both surface and subsurface hydrologic systems. By incorporating 16 geomorphological, hydrological, meteorological, geological, and geophysical layers within an AHP-based framework, the analysis successfully delineates the spatial heterogeneity of recharge potential in a structurally complex arid environment. The results demonstrate that NW–SE–trending structural depressions, synclinal basins such as the Tarboul trough, and fault-linked alluvial fan systems constitute the most promising groundwater repositories due to their enhanced permeability, sediment thickness, and drainage convergence. The observed spatial association between magnetic basement lows, drainage accumulation, and Quaternary deposits suggests that structurally controlled basins provide favorable conditions for groundwater accumulation. However, groundwater occurrence is governed by the combined influence of lithology, permeability, structural connectivity, and recharge processes.

Model validation through existing well data (AUC = 80.3%), and confusion matrix metrics and sensitivity analysis using single parameter approach confirms the stability of the AHP weighting scheme and demonstrates that structural and geological factors exert the most significant influence on groundwater potential in the study area. In addition, they confirm the reliability of the identified potential zones and highlight the southern Tarboul Basin as the most suitable priority area for future groundwater development. Although there are some uncertainties that limit the analysis, such as data accuracy, climatic variability, and well data distribution, integrated surface and subsurface methods provide a robust and applicable framework for groundwater exploration. The final conceptual model offers a simplified and informative representation of the hydrogeological reality, supporting decision makers in optimizing exploration strategies, mitigating flood risk, and planning sustainable groundwater extraction. Future work should incorporate higher-resolution datasets, additional geophysical surveys, and refined hydrogeological modeling to further reduce uncertainty and strengthen groundwater resource management in similar arid coastal regions.

## Supplementary Information

Below is the link to the electronic supplementary material.

Supplementary Material 1

## Data Availability

Data sets generated during the current study are available from the corresponding author on reasonable request.
