# Supplementary material for: A GIS-based AHP approach integrating geospatial and magnetic data for groundwater potential mapping in a structurally complex arid region, Egypt
Source: Sci Rep. 2026 May 18;16:15353. doi: 10.1038/s41598-026-52393-y (PMC13184306; doi:10.1038/s41598-026-52393-y)
Supplement: Supplementary file 1 — Supplementary Material 1 [file 41598_2026_52393_MOESM1_ESM.pdf]

A GIS-based AHP Approach Integrating Geospatial and Magnetic Data for Groundwater Potential Mapping in a Structurally Complex Arid Region, Egypt

Sara Zamzam[a,*], Ethaar Gadallah[b], Ahmed Henaish[a], Ahmed M. Nosair[b]

Table S1: Summary of principal aquifer systems in the study area [26].

Aquifer System	Lithology	Hydraulic characteristics & water quality	Recharge source
**Fractured Basement**	Fractured Granitic and Metavolcanic rocks.	Transmissivity (T):98.28 to 130.4m^2/day.<br>Storage coefficients (S): 5.05 x10^{-3} to 2.1 x10^{-3}.<br>Fresh water.	Direct precipitation & sporadic floods on exposed rocks in western parts of the study area.
**Middle Miocene Carbonate**	Reefal limestone with, evaporite, clay and shale interbeds	T: 44 to 664.9 m^2/day.<br>S: 25x10^{-5}.<br>Brackish to Saline	Downward infiltration through fractures and joints of the limestone during rainfall events.
**Quaternary**	Silt, poorly sorted sand, gravel and rock fragments in the main channels and downstream of the main watersheds.	T: 17.23 to 217 m^2/day.<br>S: 9.3x10^{-5} to 6.7x10^{-3}.<br>Fresh to Brackish	Direct infiltration from rainfall and surface runoff occurred during flash floods, especially in main channels of the watersheds

A GIS-based AHP Approach Integrating Geospatial and Magnetic Data for Groundwater Potential Mapping in a Structurally Complex Arid Region, Egypt

Sara Zamzam[a,*], Ethaar Gadallah[b], Ahmed Henaish[a], Ahmed M. Nosair[b]

Table S2: The pairwise comparison matrix of the utilized layers to build the AHP model.

	Surface lineament	geology	Rainfall	slope	subsurflin	SPI mag	DD	LULC	soil	TWI	elevation	Aspect	Curvature	SPI	TRI	STI
Surface lineament	1	1.1	1.3	1.15	1.8	1.8	2	2	3	3	3	3	4	6	6	6
geology	0.91	1	1.29	1.2	1.78	1.78	1.97	1.95	3	3	3	3	4	6	6	6
Rainfall	0.77	0.78	1	1	1.6	1.6	1.65	1.65	2.9	2.9	2	2.8	3.5	5.5	5.5	5.5
slope	0.87	0.83	1	1	1.65	1.65	1.75	1.65	2.9	2.9	2.9	2.9	4	6	6	6
subsurflin	0.56	0.56	0.63	0.61	1	1	1	1	1.4	1.45	1.45	1.45	1.6	4.5	4.5	4.5
SPI mag	0.56	0.56	0.63	0.61	1	1	1	1	1.4	1.45	1.45	1.45	1.5	4.5	4.5	4.5
DD	0.5	0.51	0.61	0.57	1	1	1	1	1.4	1.4	1.4	1.4	1.5	3	3	3
LULC	0.5	0.51	0.61	0.61	1	1	1	1	1.4	1.4	1.4	1.	1.5	2.8	2.8	2.8
soil	0.33	0.33	0.34	0.34	0.71	0.71	0.71	0.71	1	1	1	1	1	2	2	2
TWI	0.33	0.33	0.34	0.34	0.69	0.69	0.71	0.71	1	1	1	1	1	2	2	2
elevation	0.33	0.33	0.36	0.34	0.69	0.69	0.71	0.71	1	1	1	1	1	1.7	1.7	1.7
Aspect	0.33	0.33	0.36	0.34	0.69	0.69	0.71	0.71	1	1	1	1	1	1.7	1.7	1.7
Curvature	0.25	0.25	0.29	0.25	0.63	0.67	0.67	0.67	1	1	1	1	1	1.7	1.7	1.7
SPI	0.17	0.17	0.18	0.17	0.22	0.22	0.33	0.36	0.5	0.5	0.59	0.59	0.59	1	1	1.2
TRI	0.17	0.17	0.18	0.17	0.22	0.22	0.33	0.36	0.5	0.5	0.59	0.59	0.59	1	1	1.2
STI	0.17	0.17	0.18	0.17	0.22	0.22	0.33	0.36	0.5	0.5	0.59	0.59	0.59	0.83	0.83	1
**SUM**	**7.742**	**7.936**	**9.287**	**8.869**	**14.905**	**14.947**	**15.89**	**15.85**	**23.9**	**24**	**24.165**	**24.16**	**28.365**	**50.23**	**50.23**	**50.8**

A GIS-based AHP Approach Integrating Geospatial and Magnetic Data for Groundwater Potential Mapping in a Structurally Complex Arid Region, Egypt

Sara Zamzam[a,*], Ethaar Gadallah[b], Ahmed Henaish[a], Ahmed M. Nosair[b]

Table S3: The available groundwater well data that recorded in the current investigation from previous studies.

Well no	X	Y	depth	Aquifer	water type	Salinity
1	33.64136827	27.41584163	42 - 53 m	local aquifer	brackish water	..
2	33.63718838	27.41507256	42 - 53 m	local aquifer	brackish water	..
3	33.64168324	27.41476531	42 - 53 m	local aquifer	brackish water	..
4	33.64191874	27.41383268	42 - 53 m	local aquifer	brackish water	..
5	33.6405525	27.41362399	42 - 53 m	local aquifer	brackish water	..
6	33.65960769	27.39339505	18 - 30 m	local aquifer	saline water	52 - 60 ms/cm
7	33.6590657	27.39225108	18 - 30 m	local aquifer	saline water	52 - 60 ms/cm
8	33.65847105	27.39125068	18 - 30 m	local aquifer	saline water	52 - 60 ms/cm
9	33.65803951	27.39067945	18 - 30 m	local aquifer	saline water	52 - 60 ms/cm
10	33.65797864	27.38943767	18 - 30 m	local aquifer	saline water	52 - 60 ms/cm
11	33.65679646	27.38863112	18 - 30 m	local aquifer	saline water	52 - 60 ms/cm
12	33.65802537	27.38829092	18 - 30 m	local aquifer	saline water	52 - 60 ms/cm
13	33.6558909	27.38935196	18 - 30 m	local aquifer	saline water	52 - 60 ms/cm
14	33.65545797	27.38854187	18 - 30 m	local aquifer	saline water	52 - 60 ms/cm
15	33.65417272	27.38840458	18 - 30 m	local aquifer	saline water	52 - 60 ms/cm
16	33.65518448	27.38753994	18 - 30 m	local aquifer	saline water	52 - 60 ms/cm
17	33.65491183	27.38668133	18 - 30 m	local aquifer	saline water	52 - 60 ms/cm
18	33.65646295	27.38653072	18 - 30 m	local aquifer	saline water	52 - 60 ms/cm
19	33.65994347	27.38684871	18 - 30 m	local aquifer	saline water	52 - 60 ms/cm
20	33.66020538	27.38589203	18 - 30 m	local aquifer	saline water	52 - 60 ms/cm
21	33.65355371	27.38324811	18 - 30 m	local aquifer	saline water	52 - 60 ms/cm
22	33.65311605	27.38162591	18 - 30 m	local aquifer	saline water	52 - 60 ms/cm
23	33.65140417	27.38172946	18 - 30 m	local aquifer	saline water	52 - 60 ms/cm
24	33.62300865	27.37602346	68 - 107 m	local aquifer (pre Miocene)	brackish water	8 - 11 ms/cm
25	33.62729932	27.37394048	68 - 107 m	local aquifer (pre Miocene)	brackish water	8 - 11 ms/cm
26	33.62825798	27.3731336	68 - 107 m	local aquifer (pre Miocene)	brackish water	8 - 11 ms/cm
27	33.6296648	27.37209538	68 - 107 m	local aquifer (pre Miocene)	brackish water	8 - 11 ms/cm
28	33.63209668	27.37065122	68 - 107 m	local aquifer (pre Miocene)	brackish water	8 - 11 ms/cm
29	33.63299367	27.3703032	68 - 107 m	local aquifer (pre Miocene)	brackish water	8 - 11 ms/cm
30	33.63220588	27.36726845	68 - 107 m	local aquifer (pre Miocene)	brackish water	8 - 11 ms/cm
31	33.63354088	27.36491201	68 - 107 m	local aquifer (pre Miocene)	brackish water	8 - 11 ms/cm
32	33.63662741	27.36570054	68 - 107 m	local aquifer (pre Miocene)	brackish water	8 - 11 ms/cm
33	33.63736802	27.36048044	68 - 107 m	local aquifer (pre Miocene)	brackish water	8 - 11 ms/cm
34	33.64058451	27.36155492	68 - 107 m	local aquifer (pre Miocene)	brackish water	8 - 11 ms/cm

35	33.64397565	27.35396265	68 - 107 m	local aquifer (pre Miocene)	brackish water	8 - 11 ms/cm
36	33.64437068	27.35288595	68 - 107 m	local aquifer (pre Miocene)	brackish water	8 - 11 ms/cm
37	33.64372412	27.35210069	68 - 107 m	local aquifer (pre Miocene)	brackish water	8 - 11 ms/cm
38	33.64251749	27.35160464	68 - 107 m	local aquifer (pre Miocene)	brackish water	8 - 11 ms/cm
39	33.6567748	27.03961443	15 m	Fractured basement	..	7.8 ms/cm
40	33.59410149	27.08306674	19 m	alluvial sediment	..	4.8 ms/cm
41	33.54446207	27.15102782	10 - 33 m	alluvial sediment	..	
42	33.34486746	27.21878537	10 m	alluvial sediment	..	2.4 ms/cm
43	33.52045443	27.27732602	150 m	alluvial sediment	..	2.1 ms/cm
44	33.6625827	27.00828192	19.5 m	Fractured basement	..	6110 mg/l
45	33.56400886	27.16158943	20.1 m	Fractured basement	..	4080 mg/l
46	33.30468948	27.05967536	4.65 m	Fractured basement	..	1920 mg/l
47	33.35304365	27.16407039	162 m	Quaternary aquifer	..	7780 mg/l
48	33.27154074	27.1439474	32.9 m	Fractured basement	..	473 mg/l
49	33.11678062	27.24914705	5.32 m	Fractured basement	..	5080 mg/l
50	33.58560659	27.32112353	30.6 m	Miocene limestone	..	9930 mg/l
51	33.42468657	27.47920651	8.98 m	Miocene limestone	..	34200 mg/l
52	33.40570806	27.47638084	7.43 m	Miocene limestone	..	33250 mg/l
53	33.29342358	27.08955566	42.4 m	Fractured basement	..	1742 mg/l
54	33.32007167	27.33251068	15.6	Fractured basement	..	967 mg/l
55	33.36498355	27.32571227	..	Fractured basement	..	6830 mg/l
56	33.66071742	27.32677672	..	Miocene limestone	..	8770 mg/l
57	33.85247	27.03986	..	..	..	..
58	33.82305	27.16243	..	..	..	..
59	33.6551	27.23	..	..	..	..
60	33.8261	27.1436	..	..	..	..
61	33.8275	27.16305	..	..	..	..
62	33.8289	27.141012	..	..	..	..
